# The impacts of nitrous oxide gas on sleep quality during alcohol withdrawal

**DOI:** 10.1186/1756-0500-4-108

**Published:** 2011-04-07

**Authors:** Tuuli Lahti, Taina Methuen, Risto Roine, Kaija-Liisa Seppä, David Sinclair, Markku Partinen, Hannu Alho

**Affiliations:** 1 Department of Mental Health and Substance Abuse Services, National Institute for Health and Welfare, Helsinki, Finland; 2 Research Unit of Substance Abuse Medicine, University of Helsinki, Finland; 3 The Hospital District of Helsinki and Uusimaa, Finland; 4 Department of General Practice, Medical School, University of Tampere, Finland; 5 Department of Neurology, University of Helsinki, Finland; 6 Helsinki Sleep Clinic, Vitalmed Research Centre, Helsinki, Finland

## Abstract

**Background:**

Poor quality of sleep among alcoholics and persons undergoing alcohol withdrawal has been described as a possible cause of alcohol relapse. It has been suggested earlier that nitrous oxide gas has a significant effect on the signs of alcohol withdrawal syndrome (AWS) and thus might be expected to reduce sleep disturbance during withdrawal. The aim of the present study was to investigate sleep quality during alcohol withdrawal, to evaluate the correlation between sleep quality and the severity of AWS and alcohol craving, and to determine if nitrous oxide treatment does counteract withdrawal's effects on the quality of sleep. Voluntary patients (n = 105) admitted to the A-Clinic detoxification center with AWS were included in the study. The AWS patients were randomly assigned to one of the following 45-minute gas treatments: (1) nitrous oxide/oxygen; (2) normal air/O_2_; and (3) medical (normal) air. The study was single-blind by design. Sleep quality was assessed after these treatments during the inpatient period; sleep time, sleep efficiency and the fragmentation of sleep were recorded by wrist-worn actigraphs. Severity of AWS was evaluated by the Clinical Institute Withdrawal Assessment of Alcohol Scale (CIWA-Ar) and that of alcohol dependence and craving by the Obsessive Compulsive Drinking Scale [OCDS] and the Severity of Alcohol Dependence Data (SADD) questionnaire.

**Results:**

The fragmentation index and the time awake while in bed were both much above the reference values for the Finnish population. These values reflect the restless and disturbed night sleep of the subjects. The only statistically significant effects between the treatment groups were found in the correlations of CIWA-Ar (severity of AWS) scores, OCDS-scores (alcohol craving) and coffee consumption, all of which were positively associated with movement time and negatively with total sleep time and sleep efficiency. The sleep quality of patients treated with nitrous oxide gas did not differ from the sleep quality of those treated with normal air.

**Conclusions:**

The severity of AWS and coffee consumption had the most significant negative impact on sleep quality. According to our results, nitrous oxide gas does not differ from placebo in its effect on sleep quality during alcohol withdrawal.

## Background

Alcohol dependence and sleep disorders are common and often seen in combination. Chronic drinking reduces sleep latency, diminishes sleep efficiency, and disrupts the sleep pattern [1]. Withdrawal from alcohol usually results in sleep disruption, especially during the first week of withdrawal [2]. Most of the investigations using polysomnographic methods (PSG) have shown that alcohol withdrawal syndrome (AWS) is associated with long sleep, frequent awakenings and reduced slow wave sleep [3-5].

The alterations in sleep quality seen in association with AWS can last for several months [6]. If, after a period of abstinence, sleep disturbances remain, the probability of relapse increases [7]. Clark and co-workers [8] reported that subjects who relapsed had shortened rapid eye movement (REM) sleep latency, increased percentage of REM sleep and increased REM density. In the later investigation the increased REM density was successfully used as a predictor for relapse among alcoholic subjects with a secondary diagnosis of depression [4].

Sleep parameters can vary significantly between individuals. Sleep quality has commonly been assessed by using PSG and a variety of subjective self-report questionnaires. An alternative approach to PSG is to study sleep quality with wrist-worn accelometers, producing records called "actigraphs". Several studies have confirmed that actigraphy is a valid method for estimating sleep when compared to PSG [9]. Actigraphy is a useful method for investigating group differences and sleep pattern variation over time, with many benefits over PSG. The wrist-worn actigraph is a small device and thus less disturbing than classical PSG equipment. Accelometers can be used over long periods of time and during everyday activities, unlike PSG, which is usually recorded in a sleep laboratory over one or two nights. Results from actigraphic recordings also correlate well with measurements of melatonin and core body temperature rhythms. Actigraphs allow acceptable monitoring of the sleep-wake cycle, circadian rhythms and quality of sleep, and they have been used in several investigational areas [10-14], including alcohol research [14].

The treatment of AWS has been traditionally based on the application of a sedative medication such as benzodiazepine. Over a decade ago, it was suggested that nitrous oxide gas is also effective in the treatment of AWS [14-18], but this practice remains controversial. Support for using nitrous oxide was based on uncontrolled trials, while our recent placebo-controlled trial showed no differences in scores on the Clinical Institute Withdrawal Assessment of Alcohol Scale (CIWA-Ar) or in the use of benzodiazepines between subjects treated with nitrous oxide and those administered a placebo [19,20].

The mechanism of action of nitrous oxide is not known, but it has been speculated that it could activate melatonin release [21] through a direct interaction with opioid receptors. Pharmacological application of melatonin has resulted in a sleep-inducing effect and improvement in sleep quality [22]. Moreover melatonin levels in subjects with AWS have been reported to be lower than in controls [23]. If N_2_O could induce melatonin release, a possible improvement in sleep quality would be expected. Examining the effects of nitrous oxide on the changes in sleep parameters provides another independent means for determining whether the gas is capable of reversing the effects of alcohol withdrawal. The present investigation evaluates sleep quality during alcohol withdrawal and its relation to various patient characteristics. The effect of nitrous oxide on sleep quality among subjects with AWS was also assessed. The present study is a complement to the previous work of the same team [19,20].

## Methods

### Subjects

Each patient who sought treatment for alcohol withdrawal symptoms at the A-Clinic detoxification center in Leppävaara (Espoo, Finland) was interviewed. The first 105 voluntary inpatients (80 men and 25 women) meeting the study criteria were included in the study. All the participants gave informed consent. The study protocol was approved by the Helsinki University Central Hospital Ethical Committee and the study was conducted according to the International Conference on Harmonization's Good Clinical Practice Guidelines and the Declaration of Helsinki 1996. The National Agency for Medicines of Finland was notified of the study.

The inclusion criteria were age between 18 and 60 years (mean age was 44 for males, and 43 for females), and the presence of alcohol withdrawal symptoms. Exclusion criteria were an alcohol breath concentration higher than 1 μg/ml, a serious or unstable medical condition, current psychiatric disorders, and current use of psychotropic medications, previous N_2_O treatment, and recent serious convulsions during alcohol withdrawal.

### Study protocol

Subjects were randomly assigned to one of the following 45-minute gas treatments: (1) nitrous oxide/O_2 _(O_2_; starting concentration 30%, maximum 70%, aiming at a 30% EtN_2_O concentration), (n = 35); (2) normal air/O_2 _(flow 50%/50%, final O_2 _concentration 70%), (n = 35); and (3) normal air, (n = 35). The study was single-blinded by design with the patients being unaware of which treatment they were receiving. Further clinical details for this sample and a description of the study procedure are published elsewhere [19,20].

On admission, the patients completed the Severity of Alcohol Dependence Data (SADD) questionnaire [24] and the Obsessive-Compulsive Drinking Scale (OCDS) [25] questionnaire. Directly after the treatment, the ward nurse measured the CIWA-Ar scores. The measurement was repeated at 2 hours, 4 hours, 6 hours, 16-18 hours, and 40-42 hours after treatment. When the CIWA-Ar score was higher than 10, patients were administered 10 mg diazepam (orally, in liquid form) every second hour. Temazepam (20 mg/dose) was administered at night when the patient claimed sleeplessness (20 mg/night). No other medication was allowed during the study period. Coffee consumption (cups/day) and cigarette smoking (cigarettes/day) were assessed by a daily query.

### Sleep quality

During the inpatient period (1-3 days), physical activity, sleep time, and sleep quality for each subject were recorded by a wrist-worn actigraph (Actiwatch Plus^®^, Cambridge Neurotechnology Ltd, UK) device. The resulting actigraphs provide information about the quality and amount of sleep and the activity of the subject, as well as the intensity and duration of movements over extended periods of time. Sleep-wake patterns are estimated from periods of activity and inactivity based on this movement. The subjects wore the actigraph on their non-dominant wrist at all times, except when bathing or swimming. The actigraphic data were analyzed using one minute epochs with software provided by the manufacturer (The Actiwatch Sleep Analysis software, version 3.24, Cambridge Neurotechnology Ltd, UK).

A window between 10 pm and 6 am was used to compute nocturnal sleep parameters. Subjects marked what time they went to bed in the evening and woke up in the morning by pressing the event marker of the actigraphs. The period between these two events defined the time in bed (TIB). In this study we used the following actigraphic parameters to estimate the amount and quality of sleep: total sleep time (the length of sleep as determined by the Actiwatch algorithm), time awake during night (between 10 pm and 6 am), percentage of time awake during night, sleep efficiency (the percentage of time spent asleep while in bed), number of minutes moving (total number of minutes moving during sleep time) and fragmentation index. The fragmentation index is an indicator of the restlessness of sleep. It is the sum of moving time during nighttime as a percentage compared to the percentage of immobile periods. We used a medium sensitivity threshold of accelerations of 0.05 g with one minute epochs in the data storage and analyses of the Actiwatch signal. There are no official normal values for the fragmentation index (FRI). Based on our clinical experience of several hundred actigraphic recordings in subjects affected by insomnia, other sleep disorders, and in healthy people, we defined a limit of 30 for good sleep and 40 for the upper normal fragmentation index. Among working Finnish adults, less than 50% have a FRI>30 and less than 18% have a FRI>40. The time awake during the night, as measured by actigraphy, in healthy Finnish subjects has been found to be lower than 20 minutes [12], which is in accordance with other studies [26,27].

### Statistics

All statistical analyses were performed using SPSS version 11.5 statistical package (SPSS Inc, Chicago, IL). Differences in the sleeping variables were analyzed using the non-parametric Kruskal-Wallis one-way analysis of variance. When the Kruskal-Wallis test indicated the presence of statistically significant differences between the treatments at p < 0.05, the differences were tested using post hoc pair-wise multiple comparisons. To test for significant differences between the treatment arms in the sleeping variables, a general linear model (repeated measures) analysis and post hoc pair-wise multiple comparisons of the SPSS-package were used. In all multiple comparisons tests, the p values were adjusted using the Bonferroni method. Associations between sleep variables and explanatory variables were assessed by a forward stepwise multiple regression analysis (homoscedasticity). The following variables were included in the model: gender, age, breath alcohol concentration before treatment, total use of diazepam (mg), total use of temazepam [mg], coffee consumption (one unit is one coffee cup), CIWA-Ar, SADD, and OCDS before treatment, after 2 hours beyond the end of treatment, after 6 hours [evening one], after 16-18 hours (evening two), after 40-42 hours (evening three).

## Results

**Figure 1:**
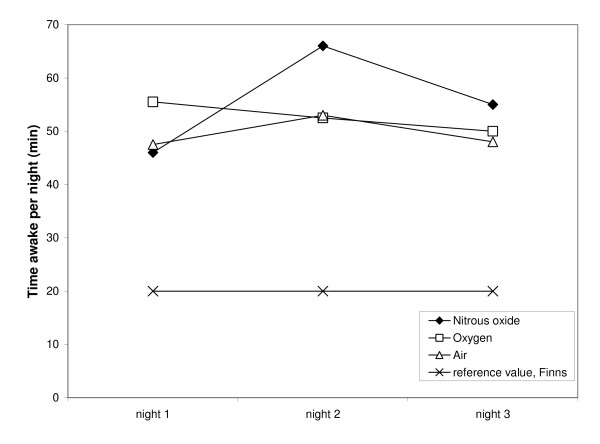
**Median values for the time awake per night in the three treatment groups during alcohol withdrawal and the normal reference value**. There were no significant differences between the groups, but all data points were far above the reference level.

**Figure 2:**
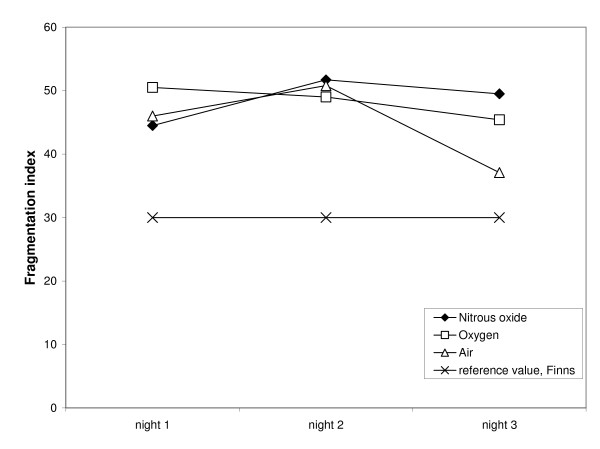
**Median sleep fragmentation indexes during three alcohol withdrawal nights for the three treatment groups and the normal reference value**. There were no significant differences between the groups.

Table 1 summarizes the median values for all sleep variables studied for the three treatments. The significance levels did not change when the use of diazepam and temazepam were included as covariates in the analysis. Table 2 shows gender and age ratios and baseline coffee consumption inside the groups (the numbers presented refer to the numbers of subjects). All groups spent more than 20 minutes awake on each of the nights (Figure 1). The sleep was disrupted, which was seen also in the high fragmentation indices (Figure 2).

**Table 1:** Median values [± semi-interquratile ranges] for sleep parameters on three nights of alcohol withdrawal for three treatment groups (the last block shows the number of subjects providing data in each condition).

Variable	night	Nitrous oxide	Oxygen	Air
**n**	1	32	30	30
	2	32	28	30
	3	31	25	30

**Total sleeping time [h]**	1	6.9 ± 0.7	6.8 ± 0.3	7.1 ± 0.5
	2	6.6 ± 0.5	6.8 ± 0.5	6.6 ± 0.7
	3	6.6 ± 0.5	6.8 ± 0.5	6.8 ± 0.5

**Time awake [min]**	1	46.0 ± 35.6	55.5 ± 13.4	47.5 ± 14.4
	2	66.0 ± 22.1	52.5 ± 31.3	53.0 ± 21.5
	3	55.0 ± 26.3	50.0 ± 25.5	48.0 ± 15.8

**% of time awake**	1	10.3 ± 7.7	12.3 ± 3.2	9.9 ± 3.2
	2	15.0 ± 4.8**	11.2 ± 6.5	11.7 ± 4.5
	3	11.6 ± 5.7	11.0 ± 5.3	10.4 ± 3.7

**Sleep efficiency**	1	86.3 ± 8.3	85.6 ± 3.8	88.3 ± 6.4
	2	82.6 ± 6.9	84.8 ± 6.8	82.6 ± 8.1
	3	82.9 ± 6.6	85.0 ± 6.5	84.9 ± 6.9

**Movement time [min]**	1	7.2 ± 4.4	9.0 ± 3.2	7.5 ± 2.6
	2	9.1 ± 3.8	8.2 ± 4.5	10.6 ± 4.9
	3	8.5 ± 4.3	8.9 ± 4.1	7.4 ± 2.5

**Fragmentation index**	1	44.5 ± 19.1	50.5 ± 13.8	46.0 ± 13.2
	2	51.7 ± 12.4	49.0 ± 13.7	50.8 ± 16.2
	3	49.5 ± 16.2	45.4 ± 9.1	37.1 ± 12.5

** Significantly different from night 1, p < 0.001.

**Table 2:** Gender, age and coffee consumption in three treatment groups.

	Nitrous oxide	Oxygen	Air	Total
**Gender**				
Male	25	21	23	69
Female	7	9	7	23

**Age**				
27-36	6	3	8	17
37-46	14	13	12	39
47-56	10	12	10	32
56 +	2	2	0	4

**Coffee consumption**				
0 cups/day	9	9	3	21
1-2 cups/day	20	19	21	60
3-5 cups/day	1	2	3	6

### Treatment differences

As shown in Table 1, the percentage of time awake in the nitrous oxide group increased significantly from the first night to the second (p < 0.001); a similar trend is seen in the time awake measure, though it failed to reach significance. There were no other significant changes within groups on different nights and also none between groups on the same nights.

### Coffee consumption

The results of the forward stepwise multiple regression analysis showed that in all groups during night 2 the sleep quality was significantly affected by coffee consumption: it was positively associated with the total minutes awake time (r^2 ^= 0.271; p = 0.016), the percentage of time awake during night time (r^2 ^= 0.263; p = 0.017), and the movement time [r^2 ^= 0.226; p = 0.029], and negatively associated with the total sleep time (r^2 ^= 0.223; p = 0.031) and the sleep efficiency (r^2 ^= 0.223; p = 0.030).

Among subjects treated with nitrous oxide, coffee consumption was negatively associated with sleep efficiency (r^2 ^= 0.210; p = 0.025) and with total sleep time (r^2 ^= 0.209; p = 0.025) during night 1 also.

### CIWA-Ar

The CIWA-Ar scores on night 1 in the group treated with treatment 2 (normal air/O_2_) were negatively associated with the total sleep time (r^2 ^= 0.408; p = 0.001) and with the sleep efficiency (r^2 ^= 0.409; p = 0.001); on night 2, CIWA-Ar scores were positively associated with total minutes awake during sleep time r^2 ^= 0.318; p = 0.005), % time awake during night-time (r^2 ^= 0.341; p = 0.003), movement time (r^2 ^= 0.273; p = 0.01) and fragmentation index (r^2 ^= 0.321; p = 0.005). In the nitrous oxide group, no significant association was observed.

### OCDS and SADD

Among subjects treated with normal air, sleep quality on night 3 correlated with OCDS and SADD scores. High OCDS scores were positively associated with total time awake (r^2 ^= 0.269; p = 0.011), % of time awake during night (r^2 ^= 0.327; p = 0.004), and movement time (r^2 ^= 0.396; p = 0.001), and were negatively associated with total sleep time (r^2 ^= 0.260; p = 0.013) and sleep efficiency (r^2 ^= 0.261; p = 0.013). SADD was positively associated with the fragmentation index (r^2 ^= 0.260; p = 0.013). In the other groups no significant associations were observed.

## Discussion

Although sleep parameters can vary between individuals, there are relatively consistent normal values: in a healthy population, subjects tend to sleep between 6 and 9 hours per night, with sleep efficiency around 85% [28]. In our study with a population of alcoholics with alcohol withdrawal symptoms, both total sleep time and sleep efficiency in all treatment groups were close to values that are considered normal for healthy subjects. This may be due to the usage of benzodiazepines for AWS; the total usage of benzodiazepines (diazepam + temazepam) during the nights was 32.2 (nitrous oxygen group), 35.7 (oxygen group) and 27.7 (air group) mg. The differences between the treatment groups are not statistically significant [20]. Both the fragmentation index and the time awake during the night were, however, much higher than the usual values that we have found in a database of several hundred actigraphic recordings made among subjects affected by insomnia and among healthy people in Finland. In our sleep clinic population we consider a fragmentation index value below 30 as an indicator of normal good sleep, and a value above 40 as abnormally high (Partinen et al. unpublished results). For the time awake during the night, values higher than 20 minutes are usually found in patients with sleep maintenance insomnia [13,26]. The median fragmentation index for our groups ranged from 35.75 to 52.70 and the time awake from 38 to 66 minutes; these high values reflect a restless and disturbed night sleep for the subjects. In summary, our results indicating poor sleep quality among subjects with AWS are in agreement with several investigations that have shown that abstinence from alcohol initially induces a degradation of sleep quality, characterized by lighter sleep, less deep sleep, and more awakenings [5,14].

High CIWA-Ar scores have been found to be negatively associated with total time awake, and positively with fragmentation index and movement time [29]. Concordantly, our multivariate analysis shows that the severity of AWS (assessed by CIWA-Ar scores) and coffee consumption are the parameters with the most significant negative impacts on sleep quality. The severity of alcohol dependence (estimated by the SADD scale) and the craving for alcohol (estimated by the Obsessive-Compulsive Drinking Scale, OCDS) also correlate negatively with sleep quality.

We found no significant differences in sleep quality between patients treated with either nitrous oxide or placebo. More specifically, there was no indication that nitrous oxide counteracted the sleep disruption during alcohol withdrawal. As shown in Figure 1, all three groups were awake more than normal on all three nights of withdrawal, and for two of the three nights, the nitrous oxide group had the largest increase. Similarly, the fragmentation index was elevated in all three groups with no tendency for nitrous oxide to reduce the disturbance (Figure 2). Rather, while the fragmentation index of the control group appears to be approaching the normal range by the third night of withdrawal, that of the nitrous oxide group is still highly elevated. This is in agreement with a previous analysis of the same sample [19] in which we found that N_2_O treatment produced signs of arousal instead of sedation.

The gold standard for sleep recording is a full-night PSG recording. Actigraphy is an appropriate method to estimate the sleep-wake cycle on an ambulatory basis [27,30,31]. However, there are some limitations to using actigraphy. First of all, without documenting the sleep- and wake times with sleep diaries, actigraphy may overestimate the sleep time. It seems that the method does not always accurately differentiate the periods of quiet wakefulness and sleep [28,30]. Thus it is important to always use sleep diaries in conjunction with actigraphy, as we did here. Second, actigraphy seems to be useful for measuring the rest-activity cycles of healthy individuals but it has to be pointed out that it is not clear whether the accuracy of the method is lower when used in clinical studies [9,27]. Thus our results may underestimate the degree of sleep disturbance in AWS. This study was designed to compare sleep between different treatment groups in AWS.

## Conclusions

In conclusion, the results of the present investigation demonstrate that subjects with AWS have a significantly deteriorated sleep quality. In an actigraphic recording this is characterized by high values for the fragmentation index and movement time, more awakenings, and low sleep efficiency. Sleep quality did not differ significantly between the three gas treatment groups. The present results complement our previous studies with the same sample [19,20], which demonstrated, using other parameters, that nitrous oxide treatment is not more effective than placebo in the treatment of AWS.

## Competing interests

The authors declare that they have no competing interests.

## Authors' contributions

All authors read and approved the final manuscript. TL participated to the drafting and revising of the manuscript; TM participated to the study planning and revising of the manuscript; RR participated in the design of the study and revision of the manuscript; KS Participated to the study planning and revising of the manuscript; DS helped with the preparation of the manuscript, the statistical interpretations, and the theoretical implications of the results; MP participated in the study planning as concerning actigraphic recordings, analysis of the data, and revising of the manuscript; HA participated to the study planning, analysis and writing of the manuscript.
